# Human Prostate Tissue MicroRNAs and Their Predicted Target Pathways Linked to Prostate Cancer Risk Factors

**DOI:** 10.3390/cancers13143537

**Published:** 2021-07-15

**Authors:** Max Enwald, Terho Lehtimäki, Pashupati P. Mishra, Nina Mononen, Teemu J. Murtola, Emma Raitoharju

**Affiliations:** 1 Pirkanmaa Hospital District, Fimlab Laboratories, and Finnish Cardiovascular Research Center Tampere, Department of Clinical Chemistry, Faculty of Medicine and Health Technology, Tampere University, 33520 Tampere, Finland; max.enwald@tuni.fi (M.E.); terho.lehtimaki@tuni.fi (T.L.); pashupati.mishra@tuni.fi (P.P.M.); nina.mononen@tuni.fi (N.M.); 2 Faculty of Medicine and Health Technology, Tampere University, 33520 Tampere, Finland; teemu.murtola@tuni.fi; 3 TAYS Cancer Center, Department of Urology, 33520 Tampere, Finland

**Keywords:** microRNA, prostate cancer, atorvastatin, risk factors

## Abstract

**Simple Summary:**

Prostate cancer is a major medical issue in men worldwide. In 2018, prostate cancer was the most frequently diagnosed cancer among men in developed countries. MicroRNAs seem to be important regulators of prostate cancer, but better understanding of their role in this disease is still needed to develop novel diagnostic tools and treatments. In this study, we aim to achieve insight on the microRNA profile of prostatic tissue environment in men with prostate cancer and to correlate this microRNA expression profile with risk factors of prostate cancer. We also examined the effects of cholesterol-lowering atorvastatin on the prostatic tissue microRNA profile. Our results provide new evidence from the effects of prostate cancer-related factors on the expression of prostate tissue microRNAs. Notably, this can reveal new pathways that may link risk factors to prostate cancer and pinpoint new therapeutic possibilities.

**Abstract:**

MicroRNAs are important in prostate cancer development, progression and metastasis. The aim of this study was to test microRNA expression profile in prostate tissue obtained from prostate cancer patients for associations with various prostate cancer related factors and to pinpoint the predicted target pathways for these microRNAs. Prostate tissue samples were obtained at prostatectomy from patients participating in a trial evaluating impact of pre-operative atorvastatin on serum prostate specific antigen (PSA) and Ki-67 expression in prostate tissue. Prostate tissue microRNA expression profiles were analyzed using OpenArray^®^ MicroRNA Panel. Pathway enrichment analyses were conducted for predicted target genes of microRNAs that correlated significantly with studied factors. Eight microRNAs correlated significantly with studied factors of patients after Bonferroni multiple testing correction. MiR-485-3p correlated with serum HDL-cholesterol levels. In atorvastatin-treated subjects, miR-34c-5p correlated with a change in serum PSA and miR-138-3p with a change in total cholesterol. In the placebo arm, both miR-576-3p and miR-550-3p correlated with HDL-cholesterol and miR-627 with PSA. In pathway analysis, these eight microRNAs related significantly to several pathways relevant to prostate cancer. This study brings new evidence from the expression of prostate tissue microRNAs and related pathways that may link risk factors to prostate cancer and pinpoint new therapeutic possibilities.

## 1. Introduction

Prostate cancer (PCa) is a major medical issue in men worldwide, and in 2018, there were 1,276,106 estimated new cases in the world. In developed countries, PCa was the most frequently diagnosed cancer among men [1]. PCa is a very heterogeneous malignancy. The 5-year survival rate for localized PCa is >99%, but for patients with metastatic PCa, it is only 30% [2]. A better understanding of the underlying mechanisms of PCa is needed in order to develop novel diagnostic tools and treatments.

MicroRNAs (miRNA, miR) are short, non-coding RNA-molecules that regulate gene expression. They mainly function through partially or completely inhibiting the translation of target mRNAs. MicroRNAs affect many cellular processes, including cell proliferation, differentiation and apoptosis, and therefore, play a key role in many syndromes and diseases including cancers [3]. MicroRNA expression profiles vary between tissues and pathological states and miRNAs could potentially serve as biomarkers in PCa [4]. They are generally stable and can be extracted from many kinds of biological samples. Examples of potential diagnostic biomarkers in PCa include miR-125b, miR221/222 and Let-7b. In addition to diagnostic biomarkers, miRNAs could also be used to assess prognosis or response to therapy [4]. MicroRNAs are also interesting targets for medical therapies, as modifying their expression levels allows for the modulation of cellular signaling pathways and networks [5].

MicroRNAs have a significant role in PCa pathogenesis, and many oncogenic and tumor-suppressive miRNAs have been identified so far [3]. They affect a variety of processes involved in cell proliferation, apoptosis, androgen receptor signaling, metastasis and epithelial mesenchymal transition (EMT) [6]. Some of the most established oncomiRs (miRNAs that promote tumor progression) in PCa include miR-1, miR-17/92a cluster, miR-21, miR-106b, miR-125b, miR-155, and miR-222/221 cluster [3,7]. There are also numerous tumor suppressive miRNAs in PCa, the most important ones including let-7 paralogs, miR-15a/16-1 cluster, miR-23b, miR-34 paralogs, miR-101, miR-126, miR-145, miR-146a, miR-200 family and miR-205 [3,7]. However, it has to be noted that these miRNAs’ expression profiles have been studied mostly in experimental models, which does not necessarily correlate with the expression profiles in human prostate tissue [7].

Known risk factors for PCa include age, ethnicity and family history. Other suggested risk factors include a high birth weight and adult height, a history of sexually transmitted infections, high plasma testosterone levels, insulin-like growth factors, obesity, hypercholesterolemia, alcohol consumption, smoking, pesticide exposure and dietary components including high calcium intake, saturated animal fat, red meat and dairy products [8,9,10]. However, evidence for these is somewhat limited and inconsistent. Protective factors most suggested by previous research include dietary components including selenium, soy, lycopene, green tea, vitamin D and E, physical activity and diabetes mellitus [8,9]. However, diabetes is controversial, as it seems to lower the risk of developing PCa, but also increases the mortality of PCa patients [11]. Drug groups that could possibly be used as chemopreventive agents for PCa include 5α-reductase inhibitors, statins, non-steroidal anti-inflammatory drugs (NSAIDs), especially aspirin and alpha-1-adrenoreceptor antagonists [12,13,14,15,16]. The use of metformin for preventative purposes is controversial [17,18].

MicroRNAs have been associated with many of these PCa-related risk factors, including insulin secretion and insulin resistance, glucose and lipids in blood, prediabetes, obesity and type 2 diabetes [19,20]. The dysregulation of lipid metabolism, which can be mediated by changes in miRNA expression, has been shown to affect PCa progression [21,22]. Lipoproteins also carry miRNAs between different tissues over circulation [23]. In addition, miRNAs have a role in androgen receptor signaling in PCa cells, thereby possibly modifying the effect of plasma androgens on these cells [24,25].

Most studies have focused on comparing the miRNA levels between PCa and normal prostate tissue and performed pathway analyses to find out the functions of the most differentially expressed miRNAs. Relating the expressed miRNA levels in prostate tissue to patients’ PCa-related characteristics in combination with the pathway analyses gives us new complementary information about the roles of the miRNAs in prostate tissue and in PCa development and can reveal new candidate miRNAs and related pathways mediating the effect of risk factors on PCa development. By analyzing the miRNA expression levels of macroscopically normal prostate tissue, rather than the tumor, we can evaluate the effect of PCa risk factors on tissue where the cellular pathways are not disrupted by the tumor and the tissue has not yet escaped the regulation of bodily signals.

The primary objective of this study is to explore the role of miRNomics in the prostatic tissue environment in men with prostate cancer and to correlate this miRNA expression profile with PCa-related risk factors. As a secondary objective, we studied the effects of a randomized intervention with cholesterol-lowering atorvastatin on the prostatic tissue miRNA profile. We found correlations between eight miRNAs and various PCa-related characteristics and risk factors. The treatment with high dose atorvastatin showed no direct effects on the miRNA expression in the prostate.

## 2. Materials and Methods

The study population comprised individuals taking part in a double-blind, placebo-controlled randomized preoperative clinical trial in which the effect of atorvastatin use in PCa patients (aged 43–75 years) scheduled for radical prostatectomy was studied [26]. The inclusion criteria were (1) histologically confirmed, previously untreated adenocarcinoma of the prostate, (2) radical prostatectomy as the first-line treatment and (3) signing of informed consent. The exclusion criteria were previous oncological treatments, previous usage of any statin, finasteride, or dutasteride within a year, liver or kidney insufficiency, previous adverse effects from cholesterol-lowering drugs and ongoing use of drugs interacting with statins [26]. In total, 158 patients were randomized to use either a daily dose of 80 mg of atorvastatin or a placebo drug for three to four weeks (median time 27 days) prior to the prostatectomy. Blood samples were taken at baseline before starting the medication and again within a week before the surgery. From the blood samples, high density lipoprotein (HDL-C) cholesterol, low density lipoprotein (LDL-C) cholesterol, total cholesterol, triglycerides, creatine kinase (CK), creatinine, alanine aminotransferase (ALAT), testosterone and PSA levels were measured using routine laboratory methods in accredited hospital testing laboratory, Fimlab Laboratories (FINAS, Finnish Accreditation Service no. T043 according to standard SFS-EN ISO15189:2013). During the recruiting process, participants were interviewed about their age, height, ongoing medications, medical history and smoking habits. The weight of the participants was measured at recruitment and body mass index (BMI) was calculated as weight (kg)/(height (m))^2^.

After prostatectomy, the tumor tissues were evaluated histologically by a study pathologist for cell proliferation activity (Ki-67 index), primary PCa histological grade and the amount of inflammatory cells. All tumors were evaluated and graded according to the International Society of Urological Pathology 2005 consensus guidelines [27]. Histologically high-grade cancer is defined as a Gleason score being equal or higher than 4 + 3 or the tertiary grade having a score of 5. Histologically low-grade cancer is defined as a Gleason score being equal or lower than 3 + 4. Intraprostatic inflammation was also evaluated according to international consensus criteria [28].

Of the 158 subjects, 64 were selected for the miRNA profiling. Subjects were selected in order to form four groups of 16 subjects in each of the following: histologically high-grade PCa patients receiving atorvastatin, low-grade PCa patients receiving atorvastatin, high-grade PCa patients receiving a placebo and low-grade PCa patients receiving a placebo. Groups were matched by age, BMI and smoking habits. One subject was disqualified from the study because of a failed miRNA-expression analysis, resulting in a total number of 63 study subjects (aged 49–75). The characteristics of the population with successful miRNA profiling are described in Table 1.

### 2.1. RNA Isolation

Prostate tissue (on average 50 mg) was taken during prostatectomy operation from the macroscopically non-cancerous part of the prostate. The fresh tissue samples were immediately soaked in RNALater solution (AMBION: RNAlater stabilization solution) and isolated with Trizol reagent (Invitrogen, Carlsbad, CA, USA) and the miRNeasy Mini Kit (Qiagen, Valencia, CA, USA) containing DNase treatment. The quality as well as concentration of the RNA was evaluated spectrophotometrically (NanoDrop 1000D, Thermo Fisher Scientific, Waltham, MA, USA).

### 2.2. Quantitative microRNA Profiling

MicroRNA expression profiling was performed following standard procedures with the quantitative real-time PCR TaqMan^®^ OpenArray^®^ MicroRNA Panel (Applied Biosystems, Waltham, MA, USA) containing 758 miRNAs. The array content is derived from miRBase v14, providing close to full coverage of Sanger well-studied miRNAs. Briefly, 100 ng of RNA was used to run both A and B pools of Megaplex (Applied Biosystems) preamplification for synthesized cDNA. In the OpenArray Sample Loading Plate, 22.5 µL of each preamplified pool was mixed 1:1 with TaqMan OpenArray Real-Time PCR Master Mix. MicroRNA panels were loaded using the AccuFill System and run with the QuantStudio 12K Flex (Applied Biosystems). Primary quality control was performed with Expression Suite Software version 1.0.1. Assays with an Amplification score > 1 and Cq Confidence > 0.7 were accepted. The miRNA data were normalized with the global mean normalization approach in R language [29]. The approach involves the calculation of the normalization factor as the global mean of all expressed miRNAs per sample. MiRNAs that were expressed in more than 50% of the subjects were included for the study, resulting in 453 miRNAs.

### 2.3. Statistical Analysis

To study associations between miRNA levels and patient characteristics, miRNA ΔCt levels were correlated with the continuous variables (age, height, weight, inflammation score, Ki-67 index, total cholesterol, LDL-C, HDL-C, triglycerides, PSA, testosterone, CK, creatinine and ALAT) using the Spearman’s rank-order correlation. Comparisons between categorical variables were performed using the Mann–Whitney U test for variables with two categories and the Kruskall–Wallis H test for variables with more than two categories.

Associations were deemed significant when the Bonferroni-corrected *p*-value (pc = *p*-value times the number of miRNAs, 453) was less than 0.05 (original *p*-value < 1.10 × 10^−4^). Correlations with less than 10 subjects were discarded. For miRNAs with significant results, fold change (FC) for the miRNA was calculated with the 2^−ΔΔCt^-method, using the median of delta CT values in the placebo arm as a reference value for each miRNA. All results are presented as the associations of miRNA FCs. MicroRNAs with significant correlations and the variables they correlated with were evaluated with the Mann–Whitney U test to see if their values differed between the statin and placebo arms. MicroRNA differences between histologically high-grade and low-grade cancer subjects were also evaluated.

To account for confounding factors, a linear regression model was created for each significant miRNA. The models were adjusted with age, BMI, smoking habits, the histological grade of the tumor, the arm of the study and the variable the miRNA showed correlation with. In the regression models, nominal *p*-value < 0.05 was considered significant. Stratified analyses were performed for atorvastatin and placebo arms separately and for the whole study population. Study arm as an explanatory variable was removed from these regression models since the correlations were only observed in one arm. As PSA decreased among all high-grade cases receiving atorvastatin, we used the Mann–Whitney U-test to see if the same held true also in this subpopulation [26].

The gene targets of the miRNAs were predicted using MirGator v.3.0 [30]. Genes that were predicted with at least two target prediction programs were selected to a pathway enrichment analysis, which was conducted with DAVID Bioinformatics Resources 6.8 [31,32]. The Homo sapiens genome was selected as background and Kyoto Encyclopedia of Genes and Genomes (KEGG) as the functional annotation database. Statistical analyses of the miRNA expression data were performed with SPSS version 25 (IBM Corp. Armonk, NY, USA).

### 2.4. MiRNA Accession Numbers

The accession numbers for the miRNAs that are mentioned are the following: MI0000093 for miR-92a-1-5p, MIMAT0000431 for miR-140-5p, MI0002469 for miR-485-3p, MI0000743 for miR-34c-5p, MI0000455 for miR-138-5p, MI0003641 for miR-627, MI0003583 for miR-576-3p, MIMAT0003257 for miR-550a-3p and MI0000089 for miR-31-5p.

## 3. Results

### 3.1. Differences in Patient Characteristics between Men in the Placebo and Atorvastatin Arms of the Study

Subjects’ characteristics between the atorvastatin and placebo arms were equally distributed (Table 1). After the intervention, statistically significant differences were found in concentrations of total cholesterol, LDL-C and triglycerides between placebo and atorvastatin arms. The change in CK, total cholesterol, HDL-C, LDL-C and triglycerides also differed significantly between the study arms (Table 1). The change in PSA levels differed significantly between the statin and the placebo arm in histologically high-grade PCa subjects (Mann–Whitney U-test 55.0, *p*-value 0.028, *n* = 29). None of the studied 453 miRNAs differed significantly (Bonferroni-corrected *p*-value (p_c_) < 0.05) between histologically high-grade and low-grade PCa subjects or between the statin and placebo arms after Bonferroni correction of multiple testing. 

### 3.2. MicroRNAs Associated Significantly with Patient Characteristics and PCa Risk Factors

The prostatic expression levels of three miRNAs had significant positive correlations with patient characteristics when examining the entire study population. MiR-485-3p correlated with serum HDL-C at the first measurement (p_c_ = 0.038) and miR-92a-1-5p with height (p_c_ = 0.011) (Table 2). In addition, miR-140-5p correlated with blood CK at the second measurement (p_c_ = 0.020). All associations also remained significant in linear regression models adjusted with age, BMI, smoking, grade of the tumor and the study arm (Table 3). 

Age, weight, BMI, smoking, tumor Ki-67 index or intraprostatic inflammation did not correlate significantly with any miRNAs after Bonferroni correction. No subjects had type 1 diabetes and only four had type 2 diabetes, and therefore, no analyses could be conducted by diabetes mellitus.

### 3.3. MiRNAs Associations by Study Arm

When examining the statin and placebo arms separately, six miRNAs showed significant correlations with tumor and patient phenotype. In the atorvastatin arm, miR-34c-5p correlated negatively with change in serum PSA during the intervention, whereas miR-138-5p correlated positively with change in total serum cholesterol. In the placebo arm, miR-627 correlated with serum PSA at the second measurement, miR-576-3p and miR-550a-3p with serum HDL-C at the second measurement and miR-92a-1-5p with height as in the whole population (Table 2). Five of the six associations remained significant (nominal *p* < 0.05) also in multivariable-adjusted linear regression models. Association between MiR-576-3p and HDL-C in the placebo arm had *p*-value of 0.058 in the adjusted regression analysis (Table 3).

In addition, prostate tissue miR-31-5p correlated significantly (r = 0.98, p_c_ = 0.015) with serum testosterone concentration (in the second follow-up measurement) in the statin-receiving arm. However, this miRNA result was not included in the further pathway analyses due to a low number of statin-receiving subjects having both the particular miRNA and testosterone successfully measured (*n* = 8).

All correlations for the factors that had significant correlations with miRNAs (presented in Table 2), with non-corrected *p*-values less than 0.05 are presented in Table S1.

### 3.4. In Silico Pathway Analyses

The prostate miRNAs that showed statistically significant correlations (presented in Table 2) were selected for pathway analyses. All the target genes for miR-550a-3p were predicted by only one prediction program, and therefore, were excluded from the final pathway analysis due to lack of confidence. The amount of target genes predicted by at least two prediction programs ranged from 14 for miR-92a-1-5p to 2311 for miR-34c-5p. Pathway analysis of the 14 genes predicted for miR-92a-1-5p did not yield any statistically significant results. The largest number of significant pathways (*n* = 31) were associated with miR-34c-5p. All pathways significantly enriched by the targets of miRNAs of interest are presented in Supplementary Table S2. A maximum of 10 of the most significantly enriched pathways for each miRNA, with nominal *p*-value < 0.05, are shown in Table 4. 

Pathways in cancer associated with four significant miRNAs (miR-140, miR-34c, miR-138 and miR-627). Adrenergic signaling in cardiomyocytes also associated with four different miRNAs. Pathways that associated with three different significant miRNAs were the thyroid hormone signaling pathway, thyroid hormone synthesis, the MAPK signaling pathway, Wnt-signaling pathway, Rap1 signaling pathway, and cAMP signaling pathway. Interestingly, the MAPK signaling pathway and proteoglycans in cancer were associated with miR-485-3p, and both the RAP1 signaling pathway and prostate cancer pathway could be seen associated with miR-34c.

## 4. Discussion

Our main findings suggest that prostatic tissue miRNAs are affected by PCa-related patient characteristics, such as serum cholesterol. These miRNAs do not change by randomized, short-term high-dose intervention with atorvastatin; therefore, the effects of atorvastatin in the prostate may not be directly mediated by these miRNAs. The observed miRNA associations may be due to long-term exposure to environmental risk factors. We have also been able to provide support for some previously known functions of the miRNAs, as well as suggest a few novel mechanisms of function.

The pathway analysis revealed that multiple PCa-relevant pathways were enriched by the gene targets of the miRNAs with significant correlations. The MAPK signaling pathway is known to regulate many key processes in cells, including proliferation and differentiation. In PCa, it has been shown to affect the epithelial–mesenchymal transition during PCa progression [33]. The dysregulation of RAP1-signaling pathway is linked to tumor cell migration and invasion in many cancers, including PCa [34]. In addition, the vascular endothelial growth factor (VEGF) signaling pathway was targeted by miR-485-3p in our study. VEGF induces angiogenesis, which is crucial for PCa progression. VEGF expression also seems to correlate with the PCa aggressiveness [35].

MiR-485-3p correlated with the subjects’ baseline HDL-C level, suggesting that miR-485-3p might be more expressed in the prostate of PCa patients with higher HDL-C. One previous study showed that miR-485-3p was downregulated in PCa tissue and more so in metastatic PCa tissue when compared to normal prostate tissue, and that the miRNA had anti-tumorigenic effects on PCa cell lines [36]. This, along with the pathway analysis that connected the miRNA to the MAPK and VEGF signaling pathways as well as proteoglycans in cancer, suggests that miR-485-3p is a tumor suppressor in PCa. Much research has been carried out to identify HDL-cholesterol’s role in the development and progression of PCa, and the latest evidence suggests that high HDL-cholesterol levels could be a risk factor for PCa [10,37]. Our finding seems to be conflicting to these previous studies. It might be that the overall effect of high HDL-cholesterol levels is tumorigenic, but it also exerts tumor-suppressive functions, perhaps through miR-485-3p.

MiR-34c-5p was found to correlate negatively with the amount of change in blood PSA in the atorvastatin-receiving arm of the study. In other words, statin-receiving subjects that experienced a greater decline in PSA during the trial, had higher levels of miR-34c-5p. Atorvastatin has been previously shown to lower PSA-levels in PCa patients [38]. In our data, the change in PSA levels differed between the statin and the placebo arm only when examining subjects with high-grade cancer. Concordantly to our findings, miR-34c expression in Pca tissue has been reported to correlate inversely with blood PSA [39]. MiR-34c is a well-established tumor suppressor in Pca [39,40]. This is also supported by the pathway analysis, as it links the miRNA to the RAP1-signaling pathway and PCa. It seems that prostatic miR-34c is associated with a better PSA-lowering effect of atorvastatin use.

MiR-138-5p was found to correlate with the change in total cholesterol in the atorvastatin arm. The decline in total cholesterol was significantly greater in the statin arm compared to the placebo arm. Since a prostate tissue miRNA is unlikely to modify atorvastatin’s effect on cholesterol synthesis mainly occurring in the liver, a more probable reason for the correlation might be that the decline in cholesterol caused a decline in the miRNA also. According to previous studies, this miRNA seems to be a tumor suppressor in PCa [41]. Since cholesterol is likely important in the development of PCa, miR-138-5p may be involved in the local effect of cholesterol in the prostate [42].

We also found a positive correlation between mir-92a-1-5p and height of the patients. This connection is in line with previous studies, since height appears to be a risk factor for high-grade PCa, and miR-92a-1 has been shown to be upregulated in many cancer types, including PCa [43,44,45]. However, this has been challenged by some studies [46,47]. MiR-92a has been previously linked to skeletal development in mice and childhood obesity, suggesting that miR-92a might have a role in growth, although the mechanism behind this is unclear [48,49]. Therefore, it might be possible that in taller men, the overall expression of miR-92a-1-5p is greater, because of its involvement in growth-related processes, and therefore, it could also be more abundant in the prostate. Our results suggest that miR-92a-1-5p could be a part of a pathway connecting height into PCa development. 

Although CK is not a known or even a suggested risk factor to PCa, an interesting association between CK and miR-140-5p was observed. MiR-140-5p was found to correlate with the amount of CK in blood at the second measurement. Change in blood CK level differed significantly between the statin and placebo arms. One of atorvastatin’s most common adverse effects is myopathy, which usually first manifests as a rise in blood CK concentration [50,51]. It seems possible that miR-140 might affect the risk of statin-induced myopathy through involvement in muscle metabolism, although confirming this would require further studies.

The high-dose atorvastatin (80 mg) treatment had no direct effects on the miRNA expression in prostatic tissues; thus, the protective effects of atorvastatin are probably not mediated directly by these studied miRNAs. However, the effect of statin treatment on the lipid profile seems to affect the expression of certain miRNAs in the prostate. Therefore, the anticancer effects of atorvastatin might be at least partly mediated indirectly through the change in lipids, affecting PCa-related miRNAs.

Our study has some limitations. The study population size was relatively small (*n* = 63); thus, statistical power was low especially in stratified analysis. All men participating in the study were Caucasian; thus, generalizability of the results to other ethnicities is uncertain. The study was conducted as a post hoc analysis, which exposes it to chance findings. The highly strict Bonferroni correction was used to reduce type I error, although it might have led to increase in type II error, with some clear biological miRNA associations being statistically non-significant. Validation in an independent cohort would also have strengthened our results. The miRNA expression analyses were conducted on prostate tissues from macroscopically normal tissue areas, rather than confirmed prostate cancer areas. This limits the comparability of our results to previous studies mostly studying miRNAs from prostate cancer tissue, but also gives us the unique possibility to estimate associations of PCa risk factors and patient characteristics on the miRNA levels in a prostatic microenvironment. Most of the gene targets of the miRNAs were so far invalidated in model organisms; thus, their relevance remains to be tested in further studies.

We had quite comprehensive background information of the study patients. The laboratory tests were conducted both before and after the atorvastatin/placebo treatment, allowing for the analysis of changes due to the intervention. By comparing the miRNA levels to patients’ characteristics and running pathway analyses on them, we could evaluate the miRNAs’ function in prostatic tissue more reliably than by pathway analysis only. In addition, we performed unbiased miRNA profiling and utilized rigid correction for multiple testing; thus, our results are not affected by preselection of miRNAs.

## 5. Conclusions

In conclusion, we found correlations between eight miRNAs and various PCa-related characteristics and risk factors. Three of these correlations were found in the entire study population, whereas the other five miRNAs only correlated significantly in the stratified analyses of the atorvastatin or placebo arms. All but one of the associations remained nominally significant in regression models adjusted with various confounding factors. We also concluded that the treatment with high-dose atorvastatin showed no direct effects on the miRNA expression in the prostate. These results altogether give new evidence from the effects of PCa-related factors on the expression of prostate tissue microRNAs. Notably, this can reveal new pathways that may link risk factors to PCa and pinpoint new therapeutic possibilities.

## Figures and Tables

**Table 1:** Characteristics of the study subjects. Continuous variables are presented as mean values with standard deviations in the parentheses. Mann–Whitney U-test was used for continuous variables and χ^2^ test for categorical variables to calculate differences between atorvastatin and placebo arms of the study. Statistically significant differences (*p*-values < 0.05) between study arms are bolded.

Variables (Units)	Total	Atorvastatin	Placebo	*p*-Values for Difference
Number of subjects (*n*)	63 (100%)	32 (51%)	31 (49%)	-
Age (years)	65.3 (5.7)	65.6 (5.6)	65.0 (5.8)	0.709
Height (cm)	179.8 (5.2)	179.3 (5.5)	180.4 (4.8)	0.397
Weight (kg)	86.7 (14.5)	86.9 (14.9)	86.6 (14.4)	0.869
Body mass index (kg/m^2^)	26.8 (4.0)	27.0 (4.1)	26.6 (4.0)	0.690
Smokers (*n*)	8 (100%)	5 (63%)	3 (37%)	0.372
High grade cancer (*n*)	31 (100%)	16 (52%)	15 (48%)	0.549
Low grade cancer (*n*)	32 (100%)	16 (50%)	16 (50%)	0.549
Inflammation score	9.4 (2.9)	9.2 (2.9)	9.6 (3.0)	0.668
Ki-67 (%)	2.9 (2.1)	3.0 (2.0)	2.8 (2.2)	0.692
Cholesterol ^1^ (mmol/L)	5.3 (0.9)	5.3 (0.8)	5.4 (1.0)	0.851
Cholesterol ^2^ (mmol/L)	4.2 (1.3)	3.2 (0.8)	5.3 (0.8)	**4.934 × 10^−9^**
ΔCholesterol (mmol/L)	−1.1 (1.2)	−2.1 (0.8)	−0.1 (0.6)	**1.6142 × 10^−9^**
HDL-C ^1^ (mmol/L)	1.5 (0.4)	1.5 (0.4)	1.6 (0.4)	0.423
HDL-C ^2^ (mmol/L)	1.4 (0.4)	1.4 (0.3)	1.5 (0.5)	0.275
ΔHDL-C (mmol/L)	−0.07 (0.2)	−0.1 (0.2)	−0.001 (0.2)	**0.014**
LDL-C ^1^ (mmol/L)	3.6 (0.9)	3.5 (0.7)	3.6 (1.0)	0.971
LDL-C ^2^ (mmol/L)	2.6 (1.2)	1.6 (0.7)	3.5 (0.7)	**9.2012 × 10^−10^**
ΔLDL-C (mmol/L)	−1.0 (1.1)	−1.9 (7.5)	−0.04 (0.5)	**1.2379 × 10^−9^**
Triglycerides^1^ (mmol/L)	1.2 (0.6)	1.2 (0.7)	1.2 (0.5)	0.333
Triglycerides^2^ (mmol/L)	1.0 (0.6)	1.0 (0.7)	1.1 (0.4)	**0.005**
ΔTriglycerides (mmol/L)	−0.2 (0.4)	−0.3 (0.4)	−0.1 (0.4)	**0.007**
PSA ^1^ (µg/L)	8.6 (4.5)	9.1 (4.6)	8.0 (4.4)	0.383
PSA ^2^ (µg/L)	8.2 (4.4)	8.7 (4.3)	7.7 (4.6)	0.315
ΔPSA (µg/l)	−0.4 (1.5)	−0.4 (1.1)	−0.3 (0.7)	0.395
Testosterone^1^ (nmol/L)	16.4 (5.3)	15.7 (4.3)	17.0 (6.0)	0.901
Testosterone^2^ (nmol/L)	16.4 (5.3)	15.2 (3.4)	17.3 (6.3)	0.643
ΔTestosterone (nmol/L)	0.2 (4.7)	−1.3 (4.3)	1.1 (4.9)	0.618
CK ^1^ (IU/l)	127.8 (69.2)	123.3 (76.2)	132.8 (61.6)	0.348
CK ^2^ (IU/l)	156.2 (111.8)	167.8 (102.0)	144.6 (121.5)	0.126
ΔCK (IU/l)	32.0 (95.7)	42.2 (64.4)	21.1 (121.0)	**0.031**

Abbreviations: CK = blood creatine kinase; cholesterol = serum total cholesterol; HDL-C = serum high density lipoprotein cholesterol; LDL-C = serum low density lipoprotein cholesterol; PSA = serum prostate specific antigen. Superscripts: 1 refers to the first baseline measurement and 2 to the second measurement of follow-up. Delta (Δ) refers to the change in laboratory value during follow up.

**Table 2:** Significant correlations found in the entire study population and in subgroup analyses. Correlation coefficients were calculated between the miRNAs and the characteristic presented under it in italics, using the Spearman’s rank-order correlation. *n* is the number of subjects included in each analysis.

MicroRNA Expressed in Prostate *Arm/Correlating Variable*	Correlation Coefficient	Nominal *p*-Value	Bonferroni-Corrected *p*-Value	*n*
**Entire population**				
miR-92a-1-5p Height (cm)	0.54	2.4 × 10^−5^	0.011	54
miR-140-5p Creatine kinase (second measurement)	0.51	4.4 × 10^−5^	0.020	58
miR-485-3p HDL-C (first measurement)	0.52	8.4 × 10^−5^	0.038	51
**Atorvastatin arm**				
miR-34c-5p Change in PSA	−0.68	4.9 × 10^−5^	0.022	29
miR-138-5p Change in total cholesterol	0.67	6.6 × 10^−5^	0.030	29
**Placebo arm**				
miR-627 PSA (second measurement)	0.74	5.7 × 10^−5^	0.026	23
miR-576-3p HDL-C (second measurement)	0.68	6.2 × 10^−5^	0.028	28
miR-550a-3p HDL-C (second measurement)	0.89	9.2 × 10^−5^	0.042	12
miR-92a-1-5p Height (cm)	0.69	9.4 × 10^−5^	0.043	26

**Table 3:** Statistics from the regression models of the miRNAs with significant correlations. The models included corresponding miRNA as the dependent variable and age, BMI, smoking habits, Gleason grade of the tumor and the arm of the study as explanatory variables. In stratified analyses, the study arm was not an explanatory variable. B is the unstandardized regression coefficient and beta is the standardized coefficient for the variable of interest. 95% CI is the confidence interval for the B-value.

MicroRNA *Correlating Variable*	B	Standard Error for B	Beta	*t*-Score	*p*-Value	95% CI
**Entire population**						
MiR-92a-1-5p Height (cm)	0.043	0.010	0.490	4.321	8.00 × 10^−5^	0.023–0.062
MiR-140-5p Creatine kinase (second measurement)	0.001	3.30 × 10^−4^	0.351	2.870	0.006	2.84 × 10^−4^–0.002
MiR-485-3p HDL-C (first measurement)	0.834	0.241	0.484	3.461	0.001	0.348–1.319
**Atorvastatin arm**						
MiR-34c-5p Change in PSA	−0.359	0.154	−0.446	−2.334	0.029	−0.678–0.041
MiR-138-5p Change in total cholesterol	1.568	0.501	0.593	3.131	0.005	0.532–2.604
**Placebo arm**						
miR-627 PSA (second measurement)	0.182	0.040	0.770	4.598	2.56 × 10^−4^	0.098–0.265
miR-576-3p HDL-C (second measurement)	0.443	0.221	0.357	1.999	0.058	−0.017–0.902
MiR-550a-3p HDL-C (second measurement)	0.555	0.084	0.483	6.579	0.001	0.348–0.761
MiR-92a-1-5p Height	0.049	0.011	0.596	4.310	0.000	0.025–0.073

**Table 4:** Pathway analyses of the miRNAs with significant results. Analysis was conducted with the genes predicted by at least two target prediction programs. A maximum of ten of the most significantly enriched pathways for each studied miRNA, with nominal *p*-value < 0.05 are shown. MiR-550a-3p was excluded from the analysis, since it had no gene targets predicted by at least two prediction programs.

Pathways for Significant miRNAs	Target Genes Enriched in Pathway	*p*-Value	Total Genes in Pathway	% of Total Genes in Pathway
**miR-92a-1-5p**				
No significant results				
**miR-140-5p**				
p53 signaling pathway	14	0.008	67	20.9
Glycosphingolipid biosynthesis-ganglio series	6	0.009	15	40.0
Insulin resistance	19	0.010	108	17.6
Adipocytokine signaling pathway	14	0.011	70	20.0
Hippo signaling pathway	24	0.011	151	15.9
Tuberculosis	27	0.012	177	15.3
Pathways in cancer	50	0.017	393	12.7
Insulin secretion	15	0.023	85	17.6
PI3K-Akt signaling pathway	44	0.025	345	12.8
Dopaminergic synapse	20	0.026	128	15.6
**miR-485-3p**				
MAPK signaling pathway	22	0.006	255	17.6
VEGF signaling pathway	8	0.020	61	8.6
Renin secretion	8	0.026	64	13.1
Proteoglycans in cancer	16	0.039	200	12.5
Non-small cell lung cancer	7	0.041	56	8.0
Wnt signaling pathway	12	0.050	138	12.5
**miR-34c-5p**				
Thyroid hormone signaling pathway	28	3.00 × 10^−4^	114	24.6
Rap1 signaling pathway	40	0.003	210	19.0
Chronic myeloid leukemia	18	0.004	72	25.0
Calcium signaling pathway	34	0.007	179	19.0
Gap junction	20	0.007	88	22.8
Prostate cancer	20	0.007	88	22.8
Adrenergic signaling in cardiomyocytes	29	0.007	146	19.9
cGMP-PKG signaling pathway	32	0.007	166	19.2
Axon guidance	26	0.007	127	20.5
Endocytosis	45	0.008	258	17.4
**miR-138-5p**				
Insulin secretion	26	5.56 × 10^−7^	85	30.6
cAMP signaling pathway	38	1.59 × 10^−4^	198	19.1
Wnt signaling pathway	29	2.43 × 10^−4^	138	21.0
Thyroid hormone signaling pathway	25	3.75 × 10^−4^	114	21.9
Acute myeloid leukemia	15	0.001	56	26.8
Pathways in cancer	60	0.001	393	15.3
Adrenergic signaling in cardiomyocytes	27	0.003	146	18.5
Endocrine and other factor-regulated calcium reabsorption	12	0.004	45	26.7
Salivary secretion	18	0.005	86	20.9
Axon guidance	23	0.008	127	18.1
**miR-627**				
Regulation of autophagy	9	1.13 × 10^−5^	39	23.1
PI3K-Akt signaling pathway	25	4.42 × 10^−5^	345	7.2
RIG-I-like receptor signaling pathway	10	1.56 × 10^−4^	70	14.3
Herpes simplex infection	16	2.25 × 10^−4^	183	8.7
Cytokine-cytokine receptor interaction	18	3.00 × 10^−4^	230	7.8
Cytosolic DNA-sensing pathway	9	4.41 × 10^−4^	64	14.1
Autoimmune thyroid disease	8	6.39 × 10^−4^	52	15.4
Natural killer cell mediated cytotoxicity	11	0.003	122	9.0
Hepatitis B	12	0.003	145	8.3
Toll-like receptor signaling pathway	10	0.003	106	9.4
**miR-576-3p**				
Thyroid hormone signaling pathway	13	6.02 × 10^−4^	114	11.4
Wnt signaling pathway	14	0.001	138	10.1
Signaling pathways regulating pluripotency of stem cells	12	0.010	140	8.6
AMPK signaling pathway	11	0.010	122	9.0
Thyroid hormone synthesis	8	0.011	70	11.4
TNF signaling pathway	10	0.012	106	9.4
Neurotrophin signaling pathway	10	0.024	120	8.3
Sphingolipid signaling pathway	10	0.024	120	8.3
Peroxisome	8	0.026	83	9.6
Adrenergic signaling in cardiomyocytes	11	0.032	146	7.5

## Data Availability

The datasets generated and/or analyzed in this study are available on reasonable request from the corresponding author. The data are not publicly available due to restrictions imposed by Finnish legislation.

## Supplementary Materials

The following are available online at https://www.mdpi.com/article/10.3390/cancers13143537/s1, Table S1: all correlations for the factors that had significant correlations with miRNAs with non-corrected *p*-values less than 0.05, Table S2: all pathways significantly enriched by the targets of miRNAs of interest.
